# Molecular mechanism underlying the selective attack of trehalose lipids on cancer cells as revealed by coarse-grained molecular dynamics simulations

**DOI:** 10.1016/j.bbrep.2021.100913

**Published:** 2021-01-20

**Authors:** Ryosuke Hirano, Takashi Kagamiya, Yoko Matsumoto, Tadaomi Furuta, Minoru Sakurai

**Affiliations:** a School of Life Science and Technology, Tokyo Institute of Technology, B-62 4259, Nagatsuta-cho, Midori-ku, Yokohama, 226-8501, Japan; b Division of Applied Life Science, Graduate School of Engineering, Sojo University, 4-22-1, Ikeda, Nishi-ku, Kumamoto, 860-0082, Japan

**Keywords:** Cancer therapy, Trehalosemonomyristate, Phosphatidylinositol, Phosphatidylserine, Membrane curvature, Membrane fusion

## Abstract

The present study indicated that the mixed lipid bilayer of dimyristoylphosphatidylcholine (DMPC) and trehalosemonomyristate (TreC14) interacted strongly with the plasma membrane of cancer cells, and not that of normal cells, when the composition of TreC14 was 70%, as revealed by coarse-grained molecular dynamics simulations. These results were consistent with those of previous experimental studies, indicating that DMPC/TreC14 mixed liposomes (DMTreC14) with TreC14 composition at 70% exhibited a strong anti-cancer effect without affecting normal cells. The simulations also revealed that lipids with highly hydrophilic and bulky head groups, such as TreC14, phosphatidylinositol (PI), and phosphatidylserine (PS), showed the tendency to accumulate. This caused both the DMTreC14 and cancer cell membranes to bend into large positive curvatures, resulting in tight contact between them. In contrast, no apparent interaction between the DMTreC14 and normal cell membranes was observed because PI and PS did not exist in the extracellular monolayer of the normal cell membrane.

## Highlights

● The mixed lipid bilayer (DMTreC14) of dimyristoylphosphatidylcholine (DMPC) and trehalosemonomyristate (TreC14) interacted strongly with the plasma membrane of a cancer cell, but did not interact with that of a normal cell.
● TreC14 was shown to interact preferentially with phosphatidylinositol (PI) and phosphatidylserine (PS), which existed in the extracellular side of the cancer cell.
● This interaction was inferred to cause the physical contact between DMTreC14 and the cancer cell, leading to their membrane fusion.

## Graphical abstract

**Figure:**
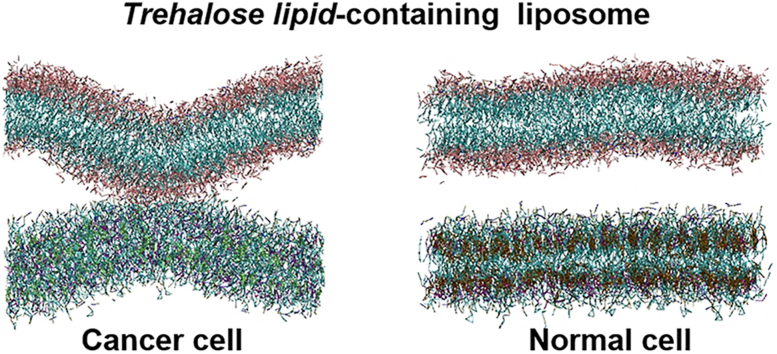

## 1 Introduction

Trehalose is a non-reducing disaccharide in which the two glucose units are linked by an α,α-1,1 glycosidic linkage. This saccharide occurs widely in nature, microorganisms, plants, and invertebrate animals [1], and is well known to act as a bioprotectant against various physico-chemical stresses [[2], [3], [4], [5], [6]], such as desiccation, heat, freezing, oxidation, and osmotic shock. The mass production of trehalose [7] brought about its extensive application in the food, cosmetic, pharmaceutical, and medicinal industries [8]. Recently, in addition to native trehalose, trehalose-conjugated derivatives have attracted much attention owing to their unique properties in biological applications [[9], [10], [11], [12], [13], [14]]. In the field of medicine, synthetically prepared trehalose fatty acid ester, α-d-glucopyranosyl-α-d-glucopyranoside monomyristate (TreC14), was found to have anti-cancer functions. For instance, hybrid liposomes composed of TreC14 and dimyristoylphosphatidylcholine (DMPC) (hereafter called DMTreC14) inhibited the growth of cells in various cancers, including human lymphoblastic leukemia and breast, hepatocellular, colon, gastric, and lung carcinoma [[15], [16], [17], [18], [19], [20]].

Cancer is the second leading cause of death globally. Currently, although chemotherapy is still considered an effective therapy for cancer in humans, most anti-cancer drugs affect healthy normal cells in the body and frequently lead to side effects. Drugs without side effects are required to attain high quality of life (QOL) for patients. DMTreC14 selectively inhibits the growth of cancer cells in a dose-dependent manner without affecting the growth of normal cells. Flow cytometric analysis revealed that apoptosis of cancer cells was induced selectively by DMTreC14 (at a particular dose), which activated the mitochondrial pathway of apoptosis via the Bcl-2 family protein (Bax) [16]. Therefore, the use of DMTreC14 as a chemotherapy agent for cancer is promising.

According to fluorescence depolarization measurements, the physico-chemical properties of DMTreC14 such as membrane fluidity change with an increase in the TreC14 content [15,16]. These membrane property changes are thought to allow selective accumulation and fusion of DMTreC14 in cancer cells [19,20]. It is likely that severe physico-chemical stresses, such as membrane fusion, act as triggers of apoptosis of cancer cells. However, how DMTreC14 selectively attacks cancer cells remains unclear.

In this study, using coarse-grained molecular dynamics (CGMD) simulations based on the MARTINI force field [21], we explored the mechanism underlying the interaction between DMTreC14 and cancer cells. DMTreC14 interacted strongly with the plasma membrane of a cancer cell, but did not interact with that of a normal cell. It was inferred that this difference was caused by the difference of the lipid compositions in the extracellular side of these cellular membranes: lipids possessing bulky and hydrophilic head groups, such as phosphatidylinositol (PI) and phosphatidylserine (PS), are abundantly present in the extracellular side of the cancer cell but absent in the case of the normal cell.

## 2 Materials and methods

### 2.1 TreC14 composition in liposome

**Fig. 1:**
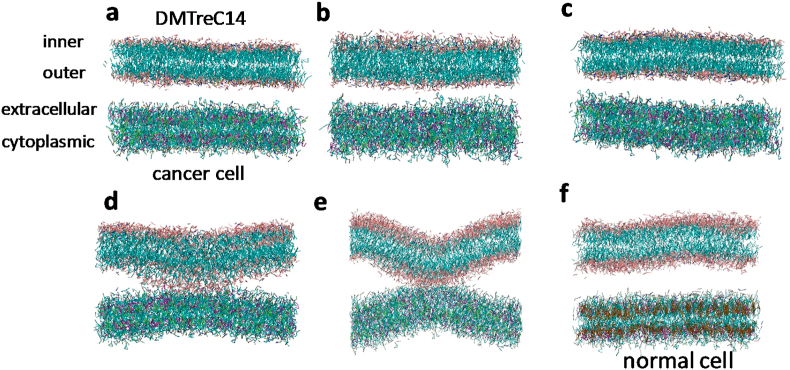
The initial structure (a) and the snapshot structures of the DMTreC14−cancer cell systems with different TreC14 compositions (b–e) and the DMTreC14 70%−normal cell system (f) at 15 μs in the CGMD simulations. TreC14 compositions are 0% (b), 30% (c), 50% (d), and 70% (e).

Here, DMTreC14 was simulated as a planar bilayer, and the molar percentage composition of TreC14 was set at 0%, 30%, 50%, or 70%. Hereafter, each system is denoted as DMTreC14 X% (X = 0, 30, 50, or 70). TreC14 molecules (Fig. S1) were distributed uniformly in both the inner and outer monolayers, where the “inner” and “outer” layers refer to the inner and outer sides of the liposome, respectively (Fig. 1a).

### 2.2 Normal and cancer cell membranes

The normal cell plasma membranes contain a variety of lipid molecules, including glycerophospholipids and sphingolipids, which are represented by sphingomyelin (SM) and cholesterol (CHL). There are various species of glycerophospholipids, resulting due to differences in their head and tail groups. According to the differences in the head groups, glycerophospholipids are divided into phosphatidylcholine (PC), phosphatidylethanolamine (PE), phosphatidylserine (PS), and phosphatidylinositol (PI). Furthermore, these species exhibit variation in their tail groups, which consist of two acyl chains that bond with the glyceryl group and vary in length and the number and locations of double bonds. Interestingly, the ratios of the component lipids of normal and cancer cell plasma membranes differ significantly. For example, comparative lipid analysis between normal murine thymocytes and leukemic (GRSL) cells indicated an increase in PE and a decrease in SM and CHL in cancer cells, compared to normal cell [22]. Additionally, a detailed analysis of the lengths of the alkyl tail chains and the number of unsaturated bonds revealed a notable decrease in the concentration of saturated tail chains in leukemic cell membranes [22].

In this study, the model normal and cancer cell membranes were constructed according to the lipid compositions of normal thymocytes and leukemic cells [22], respectively. Similar to DMTreC14, both the normal and cancer cell membranes were simulated as planar bilayers. It is well known that the lipid species in normal cell plasma membranes are asymmetrically distributed between the cytoplasmic and extracellular monolayers. The extracellular monolayer is composed mainly of PC, SM, and CHL, while the cytoplasmic monolayer is enriched with PS and PE [23]. In contrast, it has been reported that PS and PE in cancer cells are exposed to the extracellular medium [[24], [25], [26], [27]]. Thus, in a recent MD simulation of cancer cells [28], the lipid species were distributed symmetrically to emphasize PS and PE overexpression in the extracellular monolayer. Therefore, in this study, the lipid species in the cancer cells were distributed symmetrically between the extracellular and cytoplasmic monolayers. The ratio of the number of each lipid species (PC, PE, PS, SM, and CHL) between the extracellular and cytoplasmic monolayers of normal cell membranes was determined according to the corresponding ratio in ref. 28, and PI was distributed only in the cytoplasmic monolayer. The lipid compositions of the normal and cancer cell membranes are summarized in Table S1, and one monolayer contained 521 or 519 lipid molecules. More detailed information, including their fatty acid tails, is given in Table S2.

### 2.3 Simulation system

In the initial state, the DMTreC14 and cancer cell (or normal cell) bilayers were arranged at a separation of 1.8 nm in a box with dimensions 17 nm × 17 nm × 18 nm (or 16 nm × 16 nm × 18 nm). The empty space in the box was filled with 150 mM NaCl aqueous solution and counterions (Na^+^). In the DMTreC14-cancer cell membrane system, 17951 water molecules with 199 Na^+^ and Cl^−^ ions each and 128 counterions were present, while in the DMTreC14-normal cell membrane system, 15936 water molecules with 177 Na^+^ and Cl^−^ ions each and 96 counterions were present. The other simulation details are provided in the supporting information.

## 3 Results and discussion

The snapshot structures of the DMTreC14−cancer cell systems with different TreC14 compositions and the DMTreC14 70%−normal cell system at 15 μs in the CGMD simulations are shown in Fig. 1b–e and 1f, respectively. Interestingly, in the DMTreC14 50% system, both the DMTreC14 and cancer cell bilayers tended to bend (Fig. 1d), while in the DMTreC14 70% system, the curvature of the two bilayers increased and eventually they came in contact with each other (Fig. 1e). This contact was maintained until at least 30 μs (data not shown). However, in the DMTreC14 0% and 30% systems, the two bilayers were maintained at a separation (Fig. 1b and c). Similarly, in the DMTreC14 70%−normal cell system, contact of the two bilayers was not observed (Fig. 1f). Movies corresponding to Fig. 1b–f, where each lipid species is colored according to the color code in Fig. 3, are given in the supporting information.

**Fig. 2:**
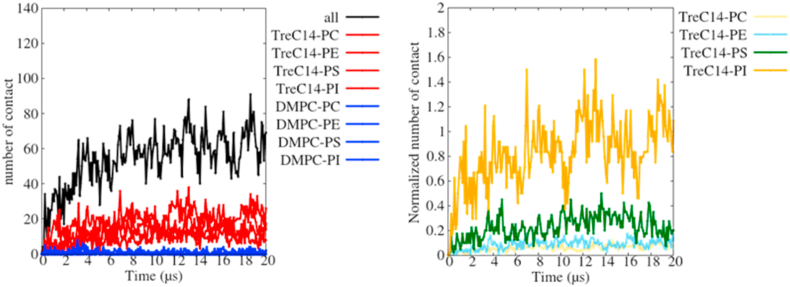
The number of contact points between the DMTreC14 and cancer cell bilayers (left), and the normalized number of TreC14-related contact points (right). In the left, the total number of contact points, black; the number of contact points between TreC14 and the different lipid species (PC, PE, PS, or PI) in the cancer cell bilayer, red; and the number of contact points between DMPC and the above-mentioned lipid species, blue, are shown. In the right, the normalized number of TreC14-related contact points with respect to the total number of each lipid species in the cancer cell bilayer (PC, yellow; PE, cyan; PS, green; PI, orange) is shown. The normalized number of contact points with respect to the number of the PI lipids is sometimes larger than one because PI sometimes interacts with the trehalose moiety of the TreC14 lipid through multiple hydrogen bonds (or multiple electrostatic interactions). (For interpretation of the references to color in this figure legend, the reader is referred to the Web version of this article.)

To examine the cause for the attractive interaction between the DMTreC14 70% and cancer cell bilayers (Fig. 1e), we counted the number of contact points for each pair of the different lipids (Fig. 2a), where the lipid pairs made contact with each other when the distance between the two beads was less than 0.5 nm. Beyond 10 μs, the number of contact points for every lipid pair became almost steady. Clearly, TreC14 (red lines) was responsible for its interaction with the different lipid species (PC, PE, PS, or PI) in the cancer cell bilayer, while DMPC (blue lines) contributed very little to its interaction with the lipid species. Fig. 2b shows the normalized number of TreC14-related contact points with respect to the total number of each lipid species in the cancer cell bilayer. Almost all PI molecules contributed to their interaction with the TreC14 molecules, even though the absolute number of PI molecules was small (Table S1). The next largest contributor was PS.

**Fig. 3:**
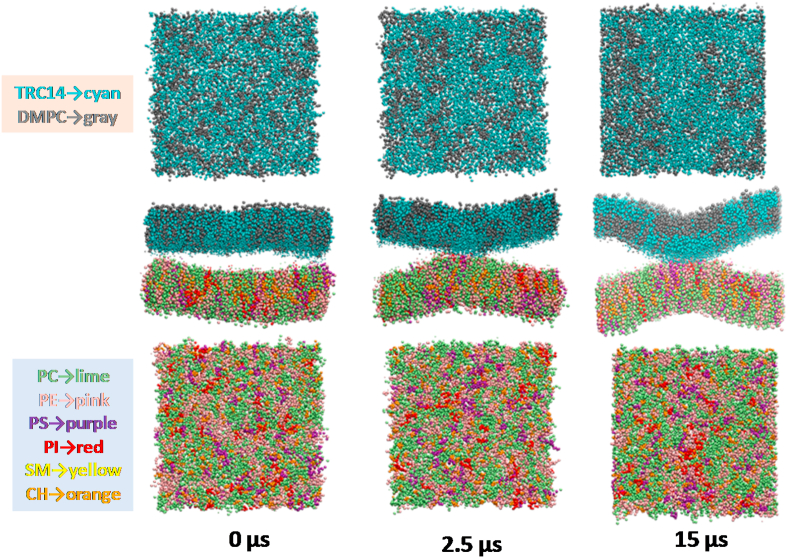
Time-dependent changes in the spatial distributions of the lipid species in the DMTreC14 and cancer cell bilayers. The upper and lower panels show the surfaces of the DMTreC14 outer monolayer and the cancer cell extracellular monolayer, respectively.

The spatial distributions of the lipid species in the two bilayers are shown in Fig. 3, where different lipid species are represented with different colors and the upper and lower panels correspond to the outer monolayer surface of the DMTreC14 membrane and the extracellular monolayer surface of the cancer cell membrane, respectively. At 0 μs, the two bilayers did not bend and the different lipid species were uniformly distributed. At 2.5 μs, the two bilayers began to bend with positive curvatures and made contact. Simultaneously, many TreC14 (cyan) lipids in the DMTreC14 outer monolayer and PI (red) and PS (purple) lipids in the cancer cell extracellular monolayer tended to accumulate along the contact region. At 15 μs, a heterogeneous distribution of lipids was clearly observed and the bilayer-bilayer contact became tighter.

The lipid distribution changes shown in Fig. 3 are interpreted as follows. First, PI and PS would interact preferentially with TreC14 because they possessed highly hydrophilic groups that enabled hydrogen bonding and electrostatic interaction with the OH groups of the trehalose moiety. As a result, PI, PS, and TreC14 accumulated and formed a domain, as shown in the snapshot at 15 μs. It is known that PI accumulation in the domain of one monolayer causes positive curvature of that monolayer because the head group of PI is bulkier than its acyl chains [29]. Similarly, TreC14 accumulation is also expected to cause positive curvature of the DMTreC14 outer monolayer due to the bulky head group of TreC14. The simultaneous positive bending of the DMTreC14 and cancer cell bilayers led to the contact between them. The interaction strength between these two bilayers depended on the TreC14 content, as shown in Fig. 1b–e. PS and PI did not exist in the extracellular monolayer of the normal cells, and thus, no apparent interaction of the normal cell membranes with DMTreC14 was observed (Fig. 1f).

Surprisingly, a good correlation between the above-mentioned TreC14-dose dependent membrane distortion and the previously reported TreC14-dose dependent inhibitory effect on cancer cell growth was observed. Based on the ascending order of the membrane distortion (DMTreC14 of 30% < 50% < 70%), the inhibitory effect evaluated by cancer cell viability (%) was elevated to 0%, 50%, and 100%, respectively [15]. In the experimental studies on DMTreC14 by Kuwabara et al. [19,20], DMTreC14 70% liposomes were fused into the cancer cell membrane. Such severe mechanical stress could induce apoptosis of the cancer cells. In our simulations, the two bilayers made stable contact but were not fused. It has been accepted that fusion of the lipid membranes occurs in several distinct steps: (1) approaching small distances, (2) local perturbation of the lipid structure and merging of the proximal monolayers, (3) stalk (hourglass-shaped hydrophobic passage) formation, (4) stalk expansion, and finally (5) pore formation [30]. Contrary to the expectations, the structure, as shown in Fig. 1e (equivalent to the structure shown in Fig. 3 at 15 μs), did not show stalk formation. According to previous theoretical studies on membrane fusion [31,32], the energy barrier that separated the above-mentioned first and third steps of membrane fusion was relatively high ≈40 *k*_*B*_*T*, where *k*_*B*_ and *T* are the Boltzmann constant and temperature, respectively. Hence, we performed the CGMD simulations at an elevated temperature (400 K) in this study. As a result, the merging of the DMTreC14 and cancer cell bilayers was observed, and a single TreC14 molecule was flipped into the cancer cell bilayer (Fig. S2), although formation of the hydrophobic passage (stalk) was not observed. In our model membranes, the proximal monolayers that faced each other were in contact with each other due to relatively strong electrostatic interactions, including those between the trehalose-inositol and trehalose-serine pairs, as shown in Fig. 2. However, the above-mentioned value of the energy barrier (40 *k*_*B*_*T*) was estimated for simple model membranes, such as DOPC and DOPE. It was likely that the activation energy required for stalk formation of realistic membranes in this study might be higher than that required for the simple model membranes. Therefore, more elongated simulation is required to observe fusion.

In conclusion, the differences in the lipid distributions of the cancer and normal cells (symmetry or asymmetry of the membrane) caused the differences in the interactions of the lipids with the DMTreC14 bilayer. The symmetric distribution of the lipids in the cancer cells allowed the PI and PS head groups to be exposed to the extracellular medium, causing their interactions with TreC14. On the other hand, the absence of such polar lipids in the extracellular monolayer of normal cells made it impossible for the lipids to interact with DMTreC14. This interpretation explained the experimental observation that DMTreC14 liposomes could be efficacious treatment agents for various cancers [[15], [16], [17], [18], [19], [20]].

## Author contributions

M.S. and T.F. designed the project. R.H. and T.K. performed the simulations and data analysis. M.S., T.F. and Y.M. reviewed and wrote the manuscript.

## Declaration of competing interest

The authors declare no conflict of interest.
