# Supplementary material for: Molecular mechanism underlying the selective attack of trehalose lipids on cancer cells as revealed by coarse-grained molecular dynamics simulations
Source: Biochem Biophys Rep. 2021 Jan 20;25:100913. doi: 10.1016/j.bbrep.2021.100913 (PMC7820381; doi:10.1016/j.bbrep.2021.100913)
Supplement: Multimedia component 6 [file mmc6.pdf]

Supporting Information for

Molecular mechanism underlying the selective attack of trehalose lipids on cancer cells as revealed by coarse-grained molecular dynamics simulations

Ryosuke Hirano[a,1], Takashi Kagamiya[a,1], Yoko Matsumoto[b], Tadaomi Furuta[a,]**, Minoru Sakurai[a,]*

[a] *School of Life Science and Technology, Tokyo Institute of Technology, B-62 4259, Nagatsuta-cho, Midori-ku, Yokohama 226-8501, Japan*

[b] *Division of Applied Life Science, Graduate School of Engineering, Sojo University, 4-22-1, Ikeda, Nishi-ku, Kumamoto 860-0082, Japan*

Details of the CGMD simulations

The CGMD simulations were performed with the Martini force field parameters (MARTINI ver 2.2) for lipids and sugars [1,2]. Water was modeled using the polarized water (PW) model [3]. The following simulation protocol was applied for every system: (i) 10,000 step steepest descent energy minimization, (ii) 30 ns equilibration under NPT conditions (310 K, 1 bar), and (iii) 15 μs production runs under the same NPT conditions (several runs were extended to 30 μs to confirm the maintenance of contact between the membranes). The production run for each system was repeated thrice. The time step was set to 20 fs. Electrostatic interactions were calculated using the reaction field method under the periodic boundary condition. The v-rescale thermostat [4] and Parrinello−Rahman barostat [5] were used to control the temperature and pressure, respectively. All CGMD simulations were performed using GROMACS 2016 (http://manual.gromacs.org/documentation/ 2016/index.html).

References

[1] S.J. Marrink, H.J. Risselada, S. Yefimov, D.P. Tieleman, A.H. de Vries, The MARTINI force field: coarse grained model for biomolecular simulations. J. Phys. Chem. B. 111 (2007) 7812−7824.

[2] López, C. A.; Rzepiela, A. J.; de Vries, A. H.; Dijkhuizen, L.; Hünenberger, P. H.; Marrink, S. J. Martini coarse-grained force field: extension to carbohydrates. J. Chem. Theory Comput. 5 (2009) 3195− 3210.

[3] Yesylevskyy, S. O.; Schäfer, L. V.; Sengupta, D.; Marrink, S. J. Polarizable water model for the coarse-grained MARTINI force field, PLoS Comput. Biol. 6 (2010) e1000810.

[4] Bussi, G.; Donadio, D.; Parrinello, M. Canonical sampling through velocity rescaling. J. Chem. Phys. 126 (2007) 014101.

[5] Parrinello, M.; Rahman, A. Polymorphic transitions in single crystals: a new molecular dynamics method. J. Appl. Phys. 52 (1981) 7182−7190.

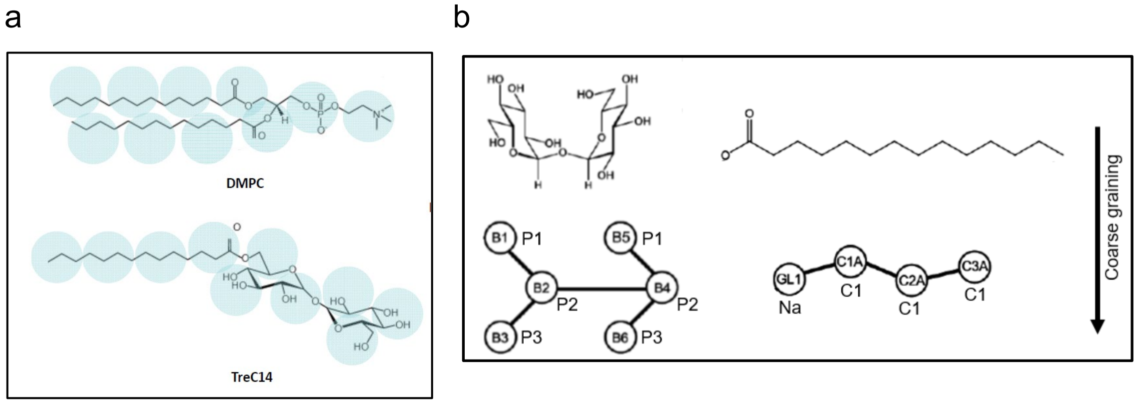

Figure S1. Chemical structures of DMPC and TreC14. (a) Chemical structures of DMPC (top) and TreC14 (bottom), and their CG representations. CG particles are represented by cyan circles. (b) CG particle types of TreC14: head and tail parts are shown in left and right, respectively.

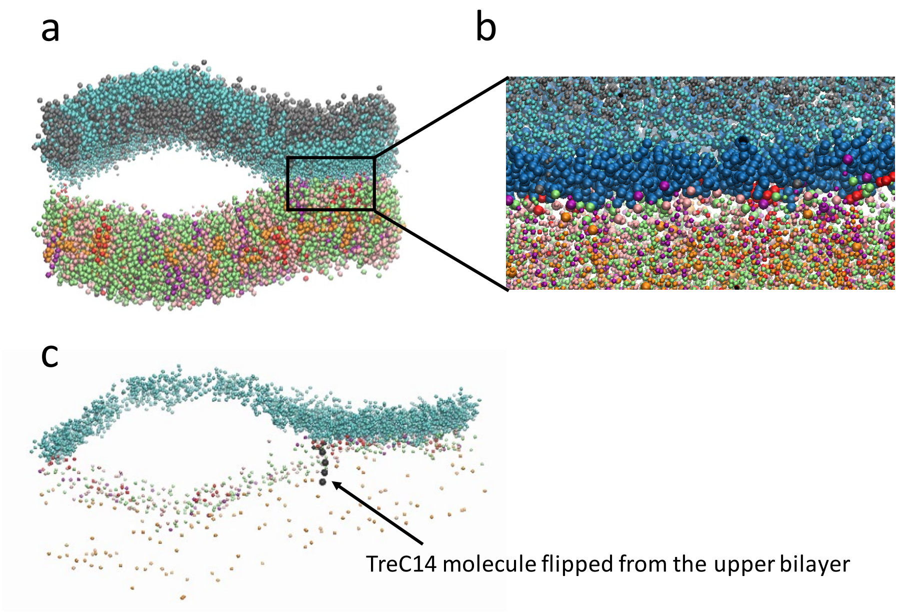

Figure S2. The snapshot structure at 15 μs in a 400 K simulation. (a) DMTreC14 and cancer cell membranes, (b) the close-up view of the contact region, and (c) the flipped TreC14 molecule. In (b), the beads of the head group are illustrated using spheres larger than those used for the fatty acid tails. The head group beads of the two proximal layers are merged. In (c), only the head group beads are shown.

Table S1. Lipid composition (number of molecules) of the extracellular and cytoplasmic monolayers of normal and cancer cell models

	Normal		Cancer	
lipid species	extracellular	cytoplasmic	extracellular	Cytoplasmic
PC	248	76	212	212
PE	30	98	120	120
PS	0	64	40	40
PI	0	32	24	24
SM	37	11	0	0
CHL	206	240	123	123
Total	521	521	519	519

Table S2. Lipid compositions of cancer and normal cells

lipid name	cancer cell	normal cell	fatty acid tails	
DPPC	0	98	1,2-dipalmitoyl	di-C16:0
MOPC	16	0	1-Margaroyl-2-oleoyl	C17:0/18:1
POPC	148	114	1-palmitoyl-2-oleoyl	C16:0/18:1
PIPC	164	48	1-palmitoyl-2-linoleoyl	C16:0/18:2
PQPC	16	16	1-palmitoyl-2-(5Z,8Z,11Z-eicosatrienoyl)	C16:0/18:3
PAPC	16	32	1-stearoyl-2-arachidonoyl	C16:0/20:4
PUPC	16	0	1-palmitoyl-2-docosahexaenoyl	C16:0/22:6
PIPC	16	0	1-palmitoyl-2-linoleoyl	C16:0/18:2
PPC	32	16	2-palmitoyl	C16:0
PIPE	32	16	1-palmitoyl-2-linoleoyl	C16:0/18:2
PAPE	48	16	1-stearoyl-2-arachidonoyl	C16:0/20:4
PUPE	48	48	1-palmitoyl-2-docosahexaenoyl	C16:0/22:6
OQPE	16	0	1-oleoyl-2-(5Z,8Z,11Z-eicosatrienoyl)	C18:1/20:3
OAPE	32	32	1-oleoyl-2-arachidonoyl	C18:1/20:4
OIPE	32	0	1-oleoyl-2-linoleoyl	C18:1/18:2
DIPE	16	0	1,2-dilinoleoyl	di-C16:2
PPE	16	16	2-palmitoyl	C16:0
POPS	32	16	1-palmitoyl-2-oleoyl	C16:0/18:1
DPPS	0	16	1,2-dipalmitoyl	di-C16:0
PIPS	32	16	1-palmitoyl-2-linoleoyl	C16:0/18:2
PAPS	16	0	1-stearoyl-2-arachidonoyl	C16:0/20:4
PUPS	0	16	1-palmitoyl-2-docosahexaenoyl	C16:0/22:6
DPPI	16	16	1,2-dipalmitoyl	di-C16:0
POPI	16	0	1-palmitoyl-2-oleoyl	C16:0/18:1
PAPI	16	16	1-stearoyl-2-arachidonoyl	C16:0/20:4
DPSM	0	48	N-stearoyl-D-erythro	C18:1/18:0
CHL	246	446	-	-

Cited from ref. 22 in text. The cancer and normal cells correspond to leukemic (GRSL) cells and normal murine thymocytes, respectively.
